# Facilitating return to work through early specialist health-based interventions (FRESH): protocol for a feasibility randomised controlled trial

**DOI:** 10.1186/s40814-015-0017-z

**Published:** 2015-06-17

**Authors:** Kathryn A Radford, Julie Phillips, Trevor Jones, Ali Gibson, Chris Sutton, Caroline Watkins, Tracey Sach, Lelia Duley, Marion Walker, Avril Drummond, Karen Hoffman, Rory O’Connor, Denise Forshaw, David Shakespeare

**Affiliations:** 1 Division of Rehabilitation and Ageing, School of Medicine, Medical School Queen’s Medical Centre, B-Floor, Nottingham, NG7 2UH UK; 2 Nottingham, UK; 3 Lancashire Clinical Trials Unit, School of Health, University of Central Lancashire, Brook Building, Room 217, Preston, PR1 2HE UK; 4 Health Economics Group, Norwich Medical School, University of East Anglia, CD Annex 1.13, Norwich Research Park, Norwich, NR4 7TJ UK; 5 Nottingham Clinical Trials Unit, Nottingham Health Science Partners, C Floor, South Block, QMC, Nottingham, NG7 2UH UK; 6 School of Health Sciences, University of Nottingham Queen’s Medical Centre (QMC), A Floor, Nottingham, NG7 2UH UK; 7 Trauma Science, Royal London Hospital, Ward 12D, Whitechapel, E1 1BB London UK; 8 Academic Department of Rehabilitation Medicine, Leeds Institute of Molecular Medicine, University of Leeds, Level D, Martin Wing, Leeds General Infirmary, Leeds, LS1 3EX UK; 9 Preston Neuro-Rehab Unit, Lancashire Teaching Hospitals NHS Trust, 32A Watling St, Preston, Lancashire PR2 8DY UK

**Keywords:** Occupational therapy, Return to work, Traumatic brain injury, Vocational rehabilitation, Feasibility, Randomised controlled trial

## Abstract

**Background:**

Over one million people sustain traumatic brain injury each year in the UK and more than 10 % of these are moderate or severe injuries, resulting in cognitive and psychological problems that affect the ability to work. Returning to work is a primary rehabilitation goal but fewer than half of traumatic brain injury survivors achieve this. Work is a recognised health service outcome, yet UK service provision varies widely and there is little robust evidence to inform rehabilitation practice. A single-centre cohort comparison suggested better work outcomes may be achieved through early occupational therapy targeted at job retention. This study aims to determine whether this intervention can be delivered in three new trauma centres and to conduct a feasibility, randomised controlled trial to determine whether its effects and cost effectiveness can be measured to inform a definitive trial.

**Methods/design:**

Mixed methods study, including feasibility randomised controlled trial, embedded qualitative studies and feasibility economic evaluation will recruit 102 people with traumatic brain injury and their nominated carers from three English UK National Health Service (NHS) trauma centres. Participants will be randomised to receive either usual NHS rehabilitation or usual rehabilitation plus early specialist traumatic brain injury vocational rehabilitation delivered by an occupational therapist. The primary objective is to assess the feasibility of conducting a definitive trial; secondary objectives include measurement of protocol integrity (inclusion/exclusion criteria, intervention adherence, reasons for non-adherence) recruitment rate, the proportion of eligible patients recruited, reasons for non-recruitment, spectrum of TBI severity, proportion of and reasons for loss to follow-up, completeness of data collection, gains in face-to-face *V*s postal data collection and the most appropriate methods of measuring primary outcomes (return to work, retention) to determine the sample size for a larger trial.

**Discussion:**

To our knowledge, this is the first feasibility randomised controlled trial of a vocational rehabilitation health intervention specific to traumatic brain injury. The results will inform the design of a definitive trial.

**Trial registration:**

The trial is registered ISRCTN Number 38581822.

**Electronic supplementary material:**

The online version of this article (doi:10.1186/s40814-015-0017-z) contains supplementary material, which is available to authorized users.

## Background

Approximately 1.4 million people in the UK sustain traumatic brain injury (TBI) each year [1] and up to 150,000 incur moderate or severe injury [2] resulting in cognitive and psychological problems that interfere with daily living activities including work. The societal cost of TBI in terms of diagnostic tests, treatment, rehabilitation, lost time at work and dependency on benefits is estimated at 2.8 billion Euros per year in Germany (price year unclear but survey conducted 2000/1) [3]. It is also a known cause of personal bankruptcy [4]. People who do not return to work are more likely to be depressed [5].

Returning to work is a primary rehabilitation goal yet reported success varies widely. Only around 41 % of TBI survivors who were working before their injury are in work at 1 and 2 years later [6]. The reasons for this are complex. Systematic reviews of factors predicting a return to work following TBI are inconclusive [7–10]. Biomedical factors such as injury severity or post injury physical or neuropsychological function alone do not fully explain work outcomes. Personal, environmental, social and organisational factors are also known to influence outcome success in supporting people with long-term conditions to return to work [11]. Whilst study heterogeneity and known difficulty in following people with TBI up over time [12] explain some of the difference in reported TBI work outcomes, inadequate rehabilitation cannot be excluded as a cause. Keeping people with TBI in work is also problematic. Many TBI survivors return prematurely but leave once the impact of the brain injury on their job is realized [13].

### What is vocational rehabilitation?

Vocational rehabilitation (VR) is defined as “whatever helps someone with a health problem to stay at, return to and remain in work” [14]. It involves helping people find work, helping those who are working but having difficulty and supporting career progression in spite of illness or disability. It is recognised as an important outcome of the UK National Health Service (NHS) health interventions [15], as a role for healthcare professionals [16] and recommended in clinical guidelines for TBI [17–19]. However, health services supporting people with TBI in returning to work are rare in the UK [20, 21]. For many people with TBI, NHS provision does not typically extend to VR. When it does, this is often towards the end of rehabilitation, after goals for independence in mobility and daily function have been achieved. People with milder head injuries and/or hidden disabilities, such as cognitive, hearing or visual impairment and those with milder TBI, are often discharged without follow-up.

There is a lack of evidence to support the effectiveness or cost effectiveness of VR for people with TBI. Whilst rehabilitation interventions for people with TBI have been evaluated using randomised controlled trials and work outcomes reported [22, 23], these were military trials and the interventions not specific to vocational rehabilitation. In a systematic review of vocational rehabilitation models, Fadyl et al. [24] identified only one randomised controlled trial (*n* = 22) that included a mixed acquired brain injury sample and only seven people with a traumatic brain injury [25].

In a single-centre cohort comparison, an early TBI specialist vocational rehabilitation intervention (ESTVR) delivered by an occupational therapist (OT), supported by a TBI case manager was compared to usual NHS rehabilitation (whatever support was available locally) and found it to be more effective (27 % more people with moderate and severe TBI in work at 12 months) at returning people with TBI to work and keeping them there 12 months after injury than usual care [26]. The mean per-patient difference in health and social care costs was only £75.00. This was because usual care participants received roughly the same amount of input but from GPs and other non-coordinated community services.

The primary focus of the ESTVR intervention was on preventing job loss by identifying people early after injury and focusing on timely returning to work with an existing employer (job retention). However, as ESTVR was an existing part of traumatic brain injury service provision in Nottingham and the intervention was delivered by a single therapist in one centre, uncertainty exists as to whether the successful outcomes were attributable to ESTVR and whether it can be delivered by therapists elsewhere.

### Aim

The primary aims are to assess the feasibility of (i) delivering early specialist traumatic brain injury vocational rehabilitation (ESTVR) in three NHS regional TBI referral centres in a way that is acceptable to people with TBI, NHS staff and employers when compared to usual NHS rehabilitation and of (ii) conducting a randomised controlled trial comparing ESTVR in addition to the usual NHS rehabilitation with usual NHS rehabilitation alone. In addition, we aim to identify the primary outcome of importance of ESTVR to service providers, service users and employers. This will enable us to determine whether a definitive evaluation trial is feasible and, if so, how its design can be optimised.

### Study objectives

In addition, we will:

1. assess the integrity of the study protocol (e.g. inclusion/exclusion criteria, staff training, adherence to intervention, and identify reasons for non-adherence);
2. estimate the recruitment rate and the proportion of potentially eligible TBI patients recruited and identify reasons non-recruitment (missed, medical, logistic, others);
3. estimate the proportion of participants lost to follow-up and the reasons for loss to follow-up;
4. determine the spectrum of TBI severity among recruits;
5. explore the views of TBI patients and staff on recruitment and the acceptability of randomisation;
6. determine the most appropriate method(s) of measuring key outcomes (return to work, retention);
7. estimate parameters necessary to calculate sample size for a larger trial (e.g. rate of return to work at 12 months in control group);
8. explore the completeness of data collection for potential primary outcome(s) for a definitive trial;
9. explore potential gains in using face-to-face rather than postal data collection; and
10. investigate how return to work is related to mood, wellbeing, function, work capacity, social participation, quality of life and carer-strain.

In a series of embedded qualitative studies, we will explore retrospectively the following items:

1. What service interventions are most valued in practice by an employee with TBI?
2. What service interventions are most valued in practice by an employer?
3. Clinical NHS staff views of the acceptability and usefulness of the ESTVR training package, including the manual and mentoring system.
4. Service user, employer and NHS staff views on factors likely to affect the ESTVR implementation and clinical delivery in the NHS.

## Methods/design

**Fig. 1:**
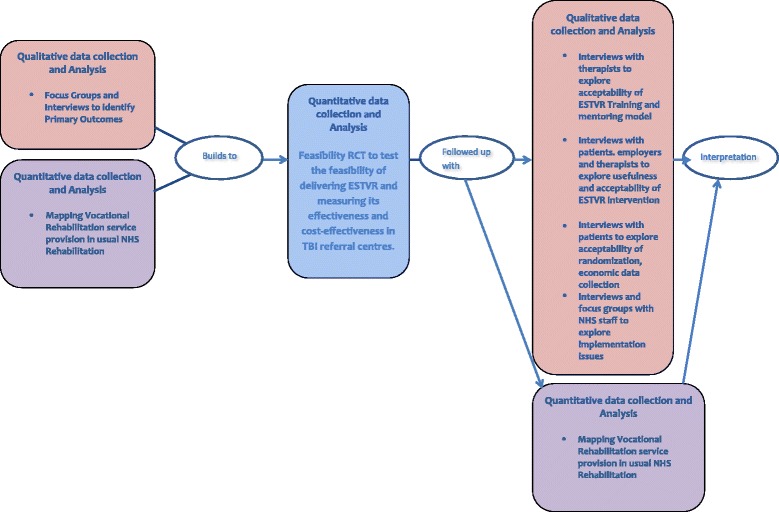
Mixed methods study configuration: interaction between feasibility trial, feasibility economic evaluation and qualitative and quantitative sub studies

Mixed methods: Feasibility individually randomised parallel-group controlled trial with embedded qualitative evaluation and feasibility economic evaluation can be seen in Fig. 1.

### Participants

Adults (aged 16 and above) living in the London, Preston and Leeds health communities and admitted for 48 h or more with new TBI and who were either in work (paid or unpaid) or in full-time education prior to their injury. Those not intending to return to work/study, unable to consent for themselves or living more than 1 h (or reasonable) travelling distance from the recruiting centre will be excluded. Nominated carers of recruited patients will also be invited to take part. Carers who are not nominated by a TBI participant will be excluded. We will attempt to include people with a language barrier or in whom English is not their first language.

### Identification and recruitment of trial participants

Potential participants will be identified by members of the existing clinical care team using existing TBI registers. The initial approach will be from a member of the patient’s usual care team, who will provide information sheets and notify the research team. The research team (research assistant or research network nurse) will inform potential participants of all aspects pertaining to participation in the study.

The University of Nottingham’s (sponsor) screening log will be used to monitor and identify recruitment against eligibility criteria and demonstrate that recruits are representative of the group as a whole. The proportion of refusals and reasons for refusal (where given) will be recorded. Every person with TBI admitted fitting the inclusion criteria during the trial recruitment period will be entered onto the screening log by the research assistant or research network nurse. Minimum data recorded will be age, gender, meeting eligibility criteria (Y/N), consented (date) or reason for non-consent.

Discharged patients will be sent a participant information sheet with covering letter from the consultant informing them about the project and stating that the researcher will contact them to ask if they are interested in taking part. If the patient expresses interest, then an appointment will be made for the researcher to visit, answer any questions and, if applicable, take informed written consent.

Consenting TBI participants will be asked to nominate a carer (spouse, partner, parent or person with whom they have most contact) during the baseline assessment visit. Carers will be sent a carer’s information sheet and covering letter from the consultant informing them about the project and stating that the researcher will contact them to ascertain their interest in taking part. Interested carers will be visited by a member of the research team to take written consent. Carers will only be recruited with consent from the TBI participant.

Completeness of carer recruitment will be verified by crosschecking TBI participants with nominated carers and the proportion of identified consenting carers recruited. This will be done by the research assistant employed in each centre.

The process for obtaining participant informed consent will be in accordance with Research Ethics Committee guidance and Good Clinical Practice. The investigator or their nominee and the participant shall both sign and date the informed consent form before the person can participate.

Baseline assessments will be completed prior to randomisation and within 8 weeks of TBI (this time frame was based on data from the original cohort comparison study where most recruitment occurred within 5 weeks of injury and because we were keen to ensure that participants received early intervention to prevent job loss). All baseline measures will be collected face-to-face by the research assistant or research nurse either in hospital or at the participant’s home if they have been discharged at the time of recruitment.

### Randomisation

Patient participants will be randomised by the research assistant using stratified randomisation (strata based on centre) via a computer-generated random allocation sequence created by Nottingham Clinical Trials Unit accessed via the web. The randomisation will be based on a computer-generated pseudo-random code using random permuted blocks of randomly varying size, created by Nottingham CTU in accordance with their standard operating procedure and held on a secure server.

The participants will be un-blinded to the intervention group allocation. Other members of the research team (chief investigator, health economist, data coordinator and trial management team) including the research assistant responsible for collecting face-to-face follow-up outcome measures and data entry staff will be blinded to group allocation. Allocation will remain concealed until all interventions are assigned and recruitment, data collection, and analyses complete.

Each participant will be assigned a trial identity code, allocated at randomisation, which will be used on case report forms, other trial documents and the electronic database. The documents and database will also use their initials and date of birth.

### Intervention and comparator

#### Early specialist traumatic brain injury vocational rehabilitation (ESTVR)

Participants (TBI patients) randomised to the intervention group will receive all usual NHS rehabilitation interventions but, in addition, will receive ESTVR (as required) targeted at job retention.

ESTVR is an early, TBI specialist, vocational rehabilitation job retention model. It was developed in Nottingham by an occupational therapist and is routinely delivered as part of usual NHS rehabilitation by the Nottingham Traumatic Brain Injury Service. It was evaluated in a single-centre cohort comparison study [27], and the results suggested a positive influence on 12-month work outcomes in those who received it. ESTVR is a case coordination model [24] based on best practice guidelines for vocational rehabilitation following acquired brain injury [19]. It is delivered by an occupational therapist, supported by a health-based case manager both of whom have knowledge and skills in working with people with a TBI and in vocational rehabilitation. Most interventions are delivered in the community.

People with TBI are identified early (at point of injury) and the intervention aims to prevent job loss. The vocational rehabilitation intervention seeks to lessen the impact of TBI by assessing the patient’s role as a worker and finding acceptable strategies to overcome problems, e.g. assessing and addressing new disabilities which might impact directly on work activities. The intervention process follows three stages, assessment, intervention, monitoring and review. Detailed assessment of the person’s occupational status and vocational aspirations and functional capacity for work, is followed by intervention to prepare the TBI person for work by providing pre-work training and establishing structured routines with gradually increasing activity levels; opportunity to practice work skills, e.g. computer use to increase concentration, cooking to practice multi-tasking. The occupational therapist liaises with employers/tutors and employment services to advise about the effects of TBI and plan and monitor-graded work return, conduct worksite visits and job evaluations, identifies the need for workplace or job adaptations and serves as the link between health and employment services to access additional support. During *monitoring and review*, progress is reviewed and ongoing advice, support and feedback provided for TBI patient, family and employer (supervisor and work colleagues as appropriate) with ongoing liaison with employment services if needed. TBI case managers coordinate the overall TBI care package and provide support, education and advice to patients, family and others, e.g. NHS staff, social services, headway and solicitors, remaining in contact with patients and families whilst there are achievable rehabilitation goals.

The intervention is tailored to individual needs according to the following menu of components:

- assessing people’s functional capacity for work;
- detailed job evaluation and safety assessment;
- liaison with employers regarding necessary accommodations (equipment and adaptations) and graduated return to work programmes;
- individual work-related goal setting and problem-solving sessions;
- partnership working with statutory and voluntary service providers such as disability employment and benefits advisors and headway;
- negotiating voluntary work placements, and
- providing information and advice to TBI patients, their families and employers and counselling.

A manualised training programme, developed in advance of the trial and based on the original Nottingham Pilot [26, 27], will be delivered centrally to occupational therapists and case managers (a nominated member of the rehabilitation team who will be trained to adopt a vocational rehabilitation case manager role) in each of the three NHS centres. The training and intervention delivery will be supported by telephone and email mentoring. Intervention delivery will be quality monitored and fidelity checks implemented to assess adherence to the ESTVR model.

#### Control: usual NHS rehabilitation

Participants allocated to the control group will avail themselves of usual health and social care services as necessary.

We will attempt to measure and describe the current focus of usual care by including resource use questions in our outcome measurement. In addition, efforts to support people with TBI in a return to work in usual NHS rehabilitation (UC) will be gathered in qualitative interviews with usual care participants.

#### Concomitant therapy

There are no known issues with the intervention and concomitant treatments, therefore no concomitant treatments will be excluded. Information on participants’ use of other community rehabilitation, social care and third-sector services will be recorded as part of the assessment of feasibility.

### Follow-up

Follow-up assessments will be completed by a research assistant masked to treatment allocation in one centre and by postal questionnaire in two centres. Steps will be taken to minimise missing data by personal contact and text messaging to prompt returns. Every attempt will be made to locate participants for follow-up.

The trial manager, or where required, a nominated designee of the sponsor (University of Nottingham), shall carry out monitoring of trial data as an ongoing activity. Entries on case report forms will be verified by inspection against the source data. A sample of case report forms (10 %) will be checked for verification of entries. In addition, the subsequent capture of data on the trial database will be checked. Where corrections are required, these will carry a full audit trail and justification.

Outcome data will be entered by a data manager blind to treatment allocation at Lancashire Clinical Trials Unit. Electronic data will be backed up every 24 h to both local and remote media in encrypted format.

### Assessment of objectives

The study adopts the National Institute for Health Research Health Technology Assessment (NIHR HTA) definition of a feasibility study [28]. The primary objective will be to assess the integrity of the study protocol to determine the feasibility of conducting a larger, appropriately powered trial. The assessment of feasibility will be determined by measuring eligible numbers, the recruitment rate per centre, the spectrum of disease among recruits, reasons for non-recruitment, compliance with vocational rehabilitation in the intervention group and with usual care in controls and the completeness of follow-up of the primary endpoint. The study will also enable us to determine whether participants can be randomised to the intervention and the likely effect on drop out of randomisation to the control group (i.e. whether patients randomised to receive no vocational rehabilitation are more likely to withdraw).

The feasibility objectives will be measured as identified in Additional file 1.

Determination of acceptability in TBI patients, staff and employers will be measured by interviewing up to 30 trial participants, 10–20 employers and the therapists providing the intervention (*n* = 4) to seek their views on the interventions (ESTVR vs UC) and in-patients and staff only, their views on recruitment and the acceptability of randomisation. We will seek to understand what service interventions are most valued in practice by an employee with TBI and which by an employer. We anticipate that we will interview between 10 and 20 employers (not all TBI participants will agree to employer contact), 15 NHS staff (5 from each centre), 4 ESTVR trained therapists and 30 trial participants (15 in each arm of the trial). It was felt that this would provide sufficient data to inform the feasibility objectives and identify issues and strategies to inform the design of the definitive trial.

The combination of the qualitative process data, plus the feasibility trial data will help to determine the integrity of the study protocol and allow us to estimate parameters necessary to calculate the sample size for a larger trial (e.g. rate of return to work at 12 months in control group).

This feasibility trial will enable us to measure recruitment, retention, the viability of delivering the intervention and measurement of the effectiveness and cost effectiveness of ESTVR plus usual NHS rehabilitation vs usual NHS rehabilitation.

As the likely primary measure of effectiveness for the main trial is work status at 12 months defined as competitive employment (full- or part-time paid work in an ordinary work setting, paid at the market rate) [29], we will record as the success criteria for the intervention at 12 months post randomization, the proportion of persons returned to and retained in:

- work in the same role with an existing employer;
- work in a different role with an existing employer;
- work with a different employer, i.e. new work, same or a different role; and
- self-employed work.

This will be collected by postal questionnaire using a series of bespoke work focussed questions.

Secondary measures of effectiveness collected at 3-, 6- and 12-month post randomisation include hospital anxiety and depression scale [30], extended activities of daily living [31], community integration questionnaire [32], EuroQol EQ-5D-3 L [33], work productivity and activity impairment questionnaire [34], carer-strain index [35], self-efficacy question from the work ability index [36] and data on the use of health and social care resources, including GP, nurse, therapists, employment services and medication use. At 12 months, the Glasgow outcome scale score will be collected as a measure of TBI recovery. This will help determine the sensitivity of measures/their value and any change related to the ESTVR intervention.

The schedule of assessments is shown in Table 1

**Table 1:** Schedule of assessments for patients and carers

Measure		Follow-up time points		
	Baseline	3 months	6 months	12 months
Schedule of assessments (patients)				
Demographic information	☑	-	-	-
Duration PTA	☑	-	-	-
GCS score	☑	-	-	-
Duration unconsciousness	☑	-	-	-
Specific VR-focused questions	☑	☑	☑	☑
EQ-5D-3 L (Euro-QOL)	☑	☑	☑	☑
Hospital anxiety and depression scale (HADS)	☑	☑	☑	☑
Nottingham extended activities of daily living (NEADL)	☑	☑	☑	☑
Community integration questionnaire (CIQ)	☑	☑	☑	☑
Resource use of health and social care	☑	☑	☑	☑
Self-efficacy—single question from work ability index	☑	☑	☑	☑
Work productivity and activity impairment questionnaire V2 (WPAI)	☑	☑	☑	☑
Glasgow outcome scale score (GOS)				☑
Schedule of assessments (carers)				
Carer strain index (CSI)	☑	☑	☑	☑
Specific impact on carer’s work questions	☑	☑	☑	☑

**Fig. 2:**
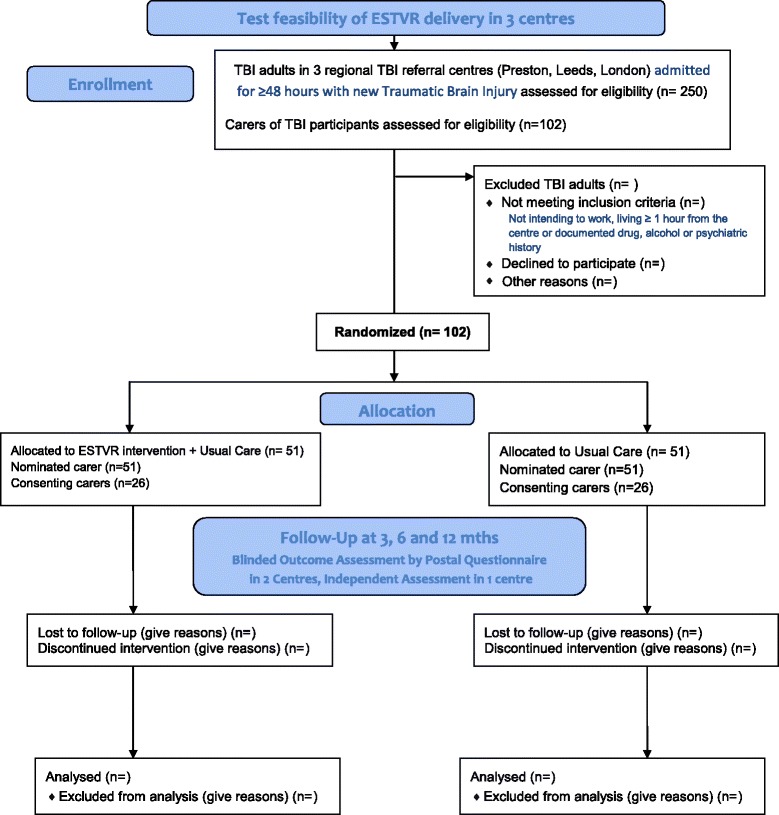
Proposed flow of participants through the feasibility trial

The proposed flow of participants through the study is shown in Fig. 2.

### Sample size justification

The sample size was based on an expectation to recruit approximately one third of patients fitting the eligibility criteria, e.g. 100 participants from 300 patients approached over 12 months. This will enable us to estimate the recruitment rate to within +/−6 % (with 95 % confidence) and the attrition rate to within +/−7 % (with 95 % confidence) (assuming attrition rate ≤15 %). The trial will recruit for 12 months. We anticipate that not all TBI participants will have or be willing to pass on carer details; however, we believe that at least 30 % of carers identified by TBI participants’ can be recruited.

### Data management

Personal data, case report forms and participant questionnaires will be treated as confidential documents and held securely in accordance with regulations at the Lancashire Clinical Trials Unit for trial participants and data from the service evaluation will be stored with the chief investigator at the University of Nottingham. Source data may include but are not limited to, consent forms, baseline data forms and current occupational therapy treatment records, audio recordings and interview transcripts from the process evaluation studies. A case report form may also completely serve as its own source data. Data will be restricted to those personnel approved by the chief or local principal investigator and recorded on the *Trial Delegation Log*.

### Analysis

Estimation of eligibility, consent and attrition rates etc. (both overall and by subgroups, e.g. site) will use descriptive statistics, supported by 95 % confidence intervals.

Effectiveness outcomes will be described at each time point and compared between groups using descriptive and inferential methods for categorical, continuous and/or ordinal health outcome measures using an intention-to-treat approach, although imputation of missing outcome data will not be performed for the primary analysis; any inferential analysis of outcomes will use 95 % confidence intervals (no *p* values will be reported). Exploratory logistic regression will be used to provide estimates of intervention effectiveness, adjusted for baseline factors previously found to be related to work return (and therefore considered to be clinically important) at 12-month post-randomisation. Investigation of the distribution of responses for health outcome measures and of patterns in work status over time will be performed to inform the design (primary outcome, follow-up duration, analysis, sample size etc.) of a future trial. Key parameters (e.g. percentage in work at 12 months in control arm) will also be estimated (with confidence intervals) to inform the design of the potential future trial. Although imputation of missing data will not be performed, we will describe the nature and extent of missing data. Relationship between return to work, mood, wellbeing, function, work capacity, social participation, quality of life and carer strain will be explored in complete case respondents.

Data will be analysed using SPSS and Stata. A detailed statistical analysis plan will be written by the trial statistician, in consultation with the Study Steering Committee and Trial Management Group, prior to un-blinding of the data.

### Embedded qualitative and quantitative sub studies

In a series of embedded qualitative and quantitative studies, we will explore the following:

- Factors that determine how much VR intervention is delivered. We will maintain detailed records of each session of the ESTVR intervention delivered by each OT using a proforma developed for use in the original cohort comparison and described elsewhere [22] and analyse the content retrospectively on a case-by-case basis to identify core components of the intervention for future trial design and replication. Features of treatment in those with successful and unsuccessful work outcomes will be identified and described using a *content of treatment* proforma [22]. Further detail about the intervention will be ascertained during participant interviews in 15 trial participants randomised to receive ESTVR.
- Practical issues relating to the deployment of the intervention will be discussed at site monitoring visits using a topic guide to include practical issues related to the screening, recruitment and consent of participants and deployment of the intervention in each group. The Nottingham therapist who developed the original ESTVR intervention and who recruited participants with TBI in the original cohort comparison study [21] will visit the newly trained ESTVR therapists and recruitment staff in each centre every 3 months. She will discuss intervention cases, review case notes and *content of treatment* proformas and monitor ESTVR fidelity by cross-referencing with data from the original study and 20 years’ clinical experience in model delivery.
- Issues relating to the training provided and required for NHS staff and participants to deploy the VR intervention. The ESTVR trained therapists views of the acceptability and usefulness of the ESTVR training, manual and mentoring will be explored in four occupational therapists trained to deliver the intervention. Their views on perceived changes in practice resulting from training and the anticipated and actual effects (including costs) of ESTVR implementation on supporting services will be ascertained. In addition, participants’, employers’ and NHS staff views of the factors likely to affect the way ESTVR vocational rehabilitation can be implemented and delivered clinically in the NHS will be explored in 15 NHS staff with a role in managing, commissioning or delivering TBI rehabilitation (five each per site) identified by local PIs and therapists involved in the ESTVR delivery, 15 TBI participants randomised to receive ESTVR and up to 10 of their employers. They will be contacted by letter and invited to participate.
- Finally, to describe the content of usual care and ESTVR in the two groups and the extent to which ESTVR occurs in usual care (the routine rehabilitation of people with TBI), we will use a questionnaire developed for a related mapping study [15] which allows components of the VR intervention delivered in any service to be mapped against a *gold standard* (best practice recommendations for vocational rehabilitation for people with long-term neurological conditions) [13]. This will enable us to identify and describe components of VR service delivery in usual care and any differences between usual care and the ESTVR model in the proposed study. VR providers in health services in each centre will be identified using data from the original mapping study plus local knowledge of PIs and therapists in each centre, to identify usual care providers. We will ask identified services to complete the questionnaire at the study outset and again at the end of the intervention period. This will capture data about the actual VR components offered by services in usual care at the study outset and allow us to describe whether usual care changed during the course of the study. As this is feasibility study, this descriptive data will allow us to characterise the variation in usual care across the three centres and pre-set criteria for planning a larger study. In addition, during participant interviews described above, the extent to which support similar to ESTVR is delivered in usual care will be explored among participants interviewed from the UC group (*n* = 15).
- To identify primary outcomes of VR that are important to service users, service providers and employers, we will conduct focus groups and interviews with people from each category. We will interview trial participants prior to randomisation to explore what outcome from vocational rehabilitation would important to them. We will also hold three focus groups, one with TBI survivors (*n* = 10) of mixed severity and time since injury identified in partnership with existing services; one with employers (*n* = 10) identified from local business networks and large employers, human resource departments, disability employment advisors, the chamber of commerce, the Federation of Small Businesses and the Employers Forum for people with disability; and one with TBI service providers in health (*n* = 10) (including doctors, nurses, and therapists). Focus groups will explore the notion of important outcomes for people with TBI following a health-based vocational rehabilitation intervention. The success criteria defined in the outcomes section of the trial plan, which are provisional and subject to change, will be presented to promote discussion about the best endpoint and nominal group technique [37] used to prioritise identified outcomes.

Using data iteratively from the qualitative interviews, focus groups and actual outcome data from our feasibility trial, we will identify *primary outcomes* of importance to explain the impact of health-based vocational rehabilitation interventions in terms of “what matters to people with TBI, what matters to TBI service providers and what matters to employers of people with TBI”.

Interviews will be digitally recorded and field notes made to capture inaudible or other contextual information. All interviews will be fully transcribed and analysed using the framework approach. The findings will inform the design of the definitive trial, the delivery of the ESTVR and the challenges likely to be faced in sustaining its delivery in the longer term.

### Health economic evaluation

This feasibility trial will allow us to determine whether we can we effectively capture economic data from people with TBI and the completeness of economic data collection needed to undertake a cost-effectiveness study comparing the overall per patient cost and effectiveness of the ESTVR, to usual care in managing working age TBI survivors. The feasibility of collecting cost and benefit data will be assessed from a health (NHS) and social care (personal social service (PSS) system) perspective to determine the frequency and costs of all NHS, social services and medication provided and from a societal perspective to determine the frequency and cost of TBI on the carers work status, the employer and government employment services, e.g. benefits and disability employment advisors.

A preliminary cost analysis will compare the overall and incremental costs for the intervention to standard practice. This sub-study will identify the resource items likely to change as a result of the ESTVR intervention, explore the practicality of collecting necessary data, and find appropriate unit cost sources to value them. In particular, we will test using bespoke patient questionnaires to capture patient costs and the ease with which patients’ self-report patient and carer costs. We will also attempt to capture the costs to employers of making *reasonable adjustment* for TBI survivors returning to work. These may include pieces of equipment or modifications to the workplace, changes to the employee’s role and responsibilities that mean other input is needed, e.g. help from employees, additional breaks, greater flexibility in terms of hours and support or supervision. However, in this feasibility study, our starting point will be to record and describe these changes and attempt to quantify them using local (data from interviews with participants and employers where reasonable adjustment has been made) and published sources.

Should data be sufficient to proceed to analysis, the cost analysis will be combined with outcome measures to perform preliminary cost-effectiveness (CEA) and cost-utility analyses (CUA). CEA and CUA produce ratio statistics in terms of cost per unit of outcome (the outcome being the percentage difference between groups of participants in work or education) and cost per quality adjusted life year (QALY), area under the curve analysis with EQ-5D-3 L values will be used to calculate QALYs. Point estimate incremental cost effectiveness ratios (ICERs) will be generated where appropriate (e.g., where the new intervention is both more expensive and more effective or less costly and less effective. Uncertainty surrounding the economic results will be explored using cost-effectiveness acceptability curves (CEACs) [38].

### Trial management and oversight

A study steering committee is in place composed of independent representatives (3:1) from the medical, academic and lay communities and representatives from the Trial Management Group. Ethical approval for this study is provided by the Northampton Research Ethics Committee (13/EM/0353) and management approval has been obtained from the trial sites.

### Safety monitoring and adverse events

As this is a feasibility trial, the side effects of the intervention are as yet unknown. We will identify these as part of this study to inform future trial design. Therefore, we propose to collect outcome data related to the intervention from participants and trial therapists including accidental injury resulting from non-compliance with equipment or work place adaptations recommended by the FRESH occupational therapists, work accidents resulting in injury requiring hospital admission, incidents of aggression (defined as excessive verbal aggression, physical aggression against objects, physical aggression against self and physical aggression against others) of the participant towards the researcher, staff or others (e.g. work colleagues), attempted suicide. All adverse outcomes will be recorded and monitored until resolution, stabilisation, or until it has been shown that the study intervention is not a likely cause.

The CI and the sponsor shall be informed immediately of any serious adverse outcomes and the seriousness and causality will be reviewed by the chief investigator in conjunction with the medical practitioner chair of the date monitoring and ethics committee which is integral to the study steering committee.

## Discussion

Work is important. It confers status and a sense of purpose and economic benefits at a personal, health and societal level. Traumatic brain injury affects people in the prime of their working life. Those who are unable to return to work face a lifetime on state benefits. The health and economic consequences of being out of work are severe and highlight the need for vocational rehabilitation to ensure those who have the capacity to work are afforded the opportunity to do so. This is now a policy imperative and an NHS outcome. However, despite widespread investigation into efforts to support people with musculoskeletal or pain-related conditions to return to or remain in work, little has been done in TBI. To our knowledge, this is the first feasibility RCT of an early health-based job retention intervention for people with TBI. If shown to be feasible, this study will provide the foundations for a future definitive trial to determine the effectiveness and cost effectiveness of early intervention to prevent job loss for people with TBI. The feasibility trial findings will be relevant to researchers and will assist service providers and commissioners in understanding the wider problem of implementing complex rehabilitation interventions in the English NHS.

We plan to disseminate our findings through presentations at national and international rehabilitation and trauma conferences and will submit for publication in peer-reviewed journals. A wider programme of dissemination will involve the use of social media, newsletters and patient and public involvement groups.

## Trial status

The trial is currently recruiting. The trial is registered with ISRCTN Number 38581822.

## Additional file

Additional file 1:
**The feasibility objectives and their measurement criteria.**

## Abbreviations

CEA: cost-effectiveness analysis
CEACs: cost-effectiveness acceptability curves
CTU: clinical trials unit
CUA: cost-utility analyses
EQ-5D-3 L: EuroQol 5D
ESTVR: early specialist TBI vocational rehabilitation
FRESH: facilitating return to work through early specialist health-based interventions
ICERS: incremental cost effectiveness ratios
ISRCTN: is a simple numeric system for the unique identification of randomised controlled trials worldwide
NHS: UK National Health Service
QALYs: quality adjusted life years
TBI: traumatic brain injury
UC: usual care
VR: vocational rehabilitation
