# Supplementary material for: Facilitating return to work through early specialist health-based interventions (FRESH): protocol for a feasibility randomised controlled trial
Source: Pilot Feasibility Stud. 2015 Jun 17;1:24. doi: 10.1186/s40814-015-0017-z (PMC5154052; doi:10.1186/s40814-015-0017-z)
Supplement: Additional file 1: — The feasibility objectives and their measurement criteria. [file 40814_2015_17_MOESM1_ESM.doc]

**Additional file 1** The feasibility objectives and their measurement criteria

| Outcome category | Objective | | Measurement process | Measurement criteria |
| --- | --- | --- | --- | --- |
| Feasibility | To assess the feasibility of conducting a randomised controlled trial comparing ESTVR in addition to usual NHS rehabilitation with usual NHS rehabilitation alone | | Feasibility trial in three NHS referral centres | Trial completion, identification of optimal design changes where needed |
| Clinical | To identify the primary outcome of importance of ESTVR to service providers, service users and employers | | Focus groups and interviews with people with TBI early and late after injury, employers and VR service providers | Identification of primary outcome |
| Process | To estimatethe recruitment rate, the proportion of potentially eligible TBI patients recruited and identify reasons non-recruitment | Eligibility | The recruitment rate and proportion of potentially eligible TBI patients (and carers) recruited and reasons for non-recruitment will be estimated using a screening log to identify recruitment against eligibility and by verifying records against site specific trauma and TBI registers  Completeness of carer recruitment will be verified by cross checking TBI participants with nominated carers against the proportion of identified consenting carers recruited | Identification of the number of potentially eligible TBI patients and carers. |
| Feasibility |  | Recruitment rate |  | Feasibility will be shown at least two TBI patients per month are recruited on average and where < two, strategies to achieve this target are identified. |
|  |  | Eligible numbers of TBI patients (and carers) recruited |  | Feasibility will be demonstrated if no fewer than 5% of TBI patients screened are recruited. |
|  |  |  |  | Feasibility will be demonstrated if not less than 25% of eligible TBI patients are recruited and where <25%, strategies are identified to achieve this. |
|  |  |  |  | Feasibility will be shown if at least 30% of eligible carers are recruited. |
| Process |  | Ease of identifying *suitable* patients |  | The number of eligible TBI patients and carers recruited  A record of reasons for non-recruitment. |
|  |  |  |  |  |
|  |  | Refusal rates |  |  |
|  |  |  |  | The number of eligible patients who refuse |
|  |  | Reasons for refusal |  | A record of the reasons for refusal where offered |
| Clinical | To determine the spectrum of TBI severity among recruits | TBI severity | TBI severity will be determined using GCS scores and length of posttraumatic amnesia | Identification of the numbers of participants with mild, moderate and severe TBI recruited. |
| Process | To estimate the proportion of participants lost to follow-up and the reasons for loss to follow-up | Compliance with trial | Loss of participants (TBI patients and carers) following recruitment and registration | The number of participants (TBI patients and carers) who are lost to follow up or withdraw from the study will be recorded.  Feasibility will be demonstrated if fewer than 40% withdraw or are lost to follow up but where>40%, if strategies to overcome identified barriers are identified. |
| Process |  | Data collection | Completeness of data collection for potential primary outcome(s) (return to work/job retention) for a definitive trial will be determined using postal questionnaires in two centres (London and Preston) and face-to-face follow-up in one (Leeds | Number of contacts (total loss v some data loss)  Number of individual outcome assessments completed (by measure)  Number of items missing within each outcome measure |
| Clinical |  | Compliance with intervention |  | The number of patients who withdraw from the intervention  Feasibility will be shown if fewer than 30 %, withdraw from the intervention or if strategies to reduce this are identified where >30 % |
| Process | To determine the most appropriate method(s) of measuring key outcomes (return to work, retention) | Work outcomes | Work outcomes will be measured using bespoke questions about work outcomes and metrics in follow-up questionnaires | Feasibility will be demonstrated if bespoke work questions are completed in 90% of completed returned questionnaires and where strategies to improve completion are identified if <90%. |
| Feasibility |  | Work status at 12 months scompetitive employment (full or part time paid work in an ordinary work setting, paid at the market rate) | Bespoke questions about work outcomes and work metrics in follow-up questionnaires | The percentage of people returned to and retained in;  a) work in the same role with an existing employer  b) work in a different role with an existing employer  c) work with a different employer i.i new work, same or a different role  d) self-employed work |
|  | To estimate parameters necessary to calculate sample size for a larger trial |  |  | The percentage of people who return to work at 12 months in the control group |
| Process | To explore the completeness of data collection for potential primary outcome(s) for a definitive trial | Loss to follow-up | The proportion of participants lost to follow-up and the reasons for loss to follow-up will be recorded | The percentage of participants lost to follow up.  Feasibility will be demonstrated if less than 30 % of participants are lost to follow-up and strategies to reduce to 20 % are identified |
| Feasibility |  | Completeness of data collection for potential primary outcome(s) |  | Feasibility will be demonstrated by less than 30 % loss to follow-up of the primary endpoint at 12 months with strategies identified to reduce to 20 % |
| Process | To explore potential gains in using face-to-face rather than postal data collection | Gains in using face-to-face rather than postal data collection | Postal questionnaires in two centres (London and Preston) and face-to-face follow-up in one (Leeds) | The number of completed patient and carer responses according to method.  The number of attempts and successful attempts to collect missing data by either method. |
| Cost |  |  |  | An estimate of the costs of face-to-face administration vs postal follow-up |
| Process | To assess the feasibility of (i) delivering ESTVR in a way that is acceptable to people with TBI, NHS staff and employers when compared to usual NHS rehabilitation | Determination of ESTVR intervention acceptability in TBI patients, staff and employers | Interviews with 30 trial participants, 10–20 employers and four therapists providing the intervention to seek their views on the interventions (ESTVR vs UC) | Feasibility will be demonstrated if most participants interviewed state that the intervention is acceptable |
|  | To explore the views of TBI patients and staff on recruitment and the acceptability of randomisation | Participants and staff views on recruitment and the acceptability of randomisation | Interviews with approximately one third (*n* = 30) of trial participants and 15 NHS staff (5 in each centre). | Feasibility will be demonstrated if most participants interviewed indicate that randomisation is acceptable |
|  |  | Acceptability and usefulness of the training | Therapists trained to deliver the ESTVR intervention (*n* = 4) will be interviewed to seek their views of the acceptability and usefulness of the training | Feasibility will be demonstrated if most of the therapists interviewed consider the training to be useful and acceptable and where not, if strategies for improvement are identified |
|  |  | Barriers to ESTVR implementation | Interviews with 4 FRESH therapists and 15 NHS staff (5 in each centre) | Identification of potential barriers to intervention delivery in trial sites and identification of strategies to address them |
| Process |  | Participant adherence/reasons for non-adherence | Interviews with therapists trained to deliver ESTVR (*n* = 4) and 15 TBI participants randomised to receive it | Identification of reasons for non-adherence |
| Process and cost |  | Trial feasibility | End of study focus groups with staff in each centre to explore practical problems in running the trial | Identification of practical problems with trial delivery and solutions to overcome them, e.g.  Centres’ willingness and capacity to recruit  Centres’ ability to honour commitment to study  Local challenges in managing the study and personnel  Utility of forms  Time taken to complete CRFs and screening logs  Problems with study processes (screening, recruitment, randomisation, data collection, entry and transfer) |
| Cost | To determine whether we can effectively capture economic data from people with TBI and the completeness of economic data collection needed to undertake a cost-effectiveness study comparing the overall per patient cost and effectiveness of the ESTVR, to usual care in managing working age TBI survivors | Practicality of collecting and valuing cost data. | Bespoke patient questionnaires to capture patient costs and the ease of self-reported patient and carer costs. | Record of changes in patient, health, social care and employment related costs resulting from TBI  Quantification of costs using (data from) local and published sources  Completeness of self-reported cost data  Identification of resource items and appropriate unit cost sources |
|  |  |  | Interviews with TBI participants and employers where reasonable adjustment has been made to capture the costs to employers of making *reasonable adjustment* for TBI | Description of reasonable adjustments  Costs of reasonable adjustments |
